# Prediction of tau accumulation in prodromal Alzheimer’s disease using an ensemble machine learning approach

**DOI:** 10.1038/s41598-021-85165-x

**Published:** 2021-03-11

**Authors:** Jaeho Kim, Yuhyun Park, Seongbeom Park, Hyemin Jang, Hee Jin Kim, Duk L. Na, Hyejoo Lee, Sang Won Seo

**Affiliations:** 1 Department of Neurology, Dongtan Sacred Heart Hospital, Hallym University College of Medicine, Hwaseong-si, Gyeonggi-do Republic of Korea; 2 Department of Neurology, Samsung Medical Center, Sungkyunkwan University School of Medicine, 81 Irwon-ro, Gangnam-gu, Seoul, 06351 Republic of Korea; 3 Department of Intelligent Precision Healthcare Convergence, Sungkyunkwan University School of Medicine, Suwon, Republic of Korea; 4 Neuroscience Center, Samsung Medical Center, Seoul, Republic of Korea; 5 Samsung Alzheimer Research Center, Samsung Medical Center, Seoul, Republic of Korea; 6 Stem Cell and Regenerative Medicine Institute, Samsung Medical Center, Seoul, Republic of Korea; 7 Department of Health Sciences and Technology, SAIHST, Sungkyunkwan University, Seoul, Republic of Korea

**Keywords:** Cognitive ageing, Neuroscience, Diseases of the nervous system, Alzheimer's disease

## Abstract

We developed machine learning (ML) algorithms to predict abnormal tau accumulation among patients with prodromal AD. We recruited 64 patients with prodromal AD using the Alzheimer’s Disease Neuroimaging Initiative (ADNI) dataset. Supervised ML approaches based on the random forest (RF) and a gradient boosting machine (GBM) were used. The GBM resulted in an AUC of 0.61 (95% confidence interval [CI] 0.579–0.647) with clinical data (age, sex, years of education) and a higher AUC of 0.817 (95% CI 0.804–0.830) with clinical and neuropsychological data. The highest AUC was 0.86 (95% CI 0.839–0.885) achieved with additional information such as cortical thickness in clinical data and neuropsychological results. Through the analysis of the impact order of the variables in each ML classifier, cortical thickness of the parietal lobe and occipital lobe and neuropsychological tests of memory domain were found to be more important features for each classifier. Our ML algorithms predicting tau burden may provide important information for the recruitment of participants in potential clinical trials of tau targeting therapies.

## Introduction

Mild cognitive impairment (MCI) refers to a transitional state between normal ageing and Alzheimer's disease (AD)^1,2^. The rapid development of molecular imaging methods has enabled the detection of amyloid-β (Aβ) using positron emission tomography (PET) in the MCI stages. Previous studies have shown that 40–60% of MCI patients are Aβ positive (Aβ+) on PET, a characteristic of prodromal AD^3–5^. Recently, these patients with prodromal AD have also been classified into fast and slow decliners according to their downstream biomarker status^6^. Especially, considering that the presence of neurofibrillary tangles (NFTs) formed by tau is a highly predictive indicator for cognitive decline^7^, it is important to develop methods to detect tau uptake in prodromal AD in vivo. In this regard, a recent AV-1451 PET study^8^, which investigated the NFT burden in the brain, reported that patients with prodromal AD exhibit in-vivo Braak stages ranging from I/II to V/VI. However, like all PET techniques, its clinical utility in medical practice has been limited because of its cost, availability, and safety, as there are risks regarding radiation exposure^9^. Regardless, the prediction of tau uptake remains an important goal, as the expectation is that future treatment strategies may target tau protein.

The exponential growth of computing power with massive data sets has led to machine learning (ML) being an alternative analytics method for clinical decision making and for searching for new relationships between disease and symptoms. Random forest (RF)^10^ and gradient boosting machine (GBM)^11^ are commonly used ML methods^12^ that have been outperformed consistently in many large-scale studies^13^. In addition, unlike other ML predictions with routinely used performance measures, tree-based ML provides clinically useful information, such as the relative importance of the clinical features and whether they are related positively or negatively. However, these interpretable ML methods have not been used for classifying tau burden in previous studies.

In a previous study, worse performance on the domain-specific neuropsychological tests was associated with a greater ^18^F-AV1451 uptake in key regions implicated in memory, visuospatial function, and language^14^. In the combined prodromal AD and AD dementia group, increased tau PET uptake and reduced cortical thickness were associated with worse performance on a variety of neuropsychological tests^15^. Altogether, these biomarkers seem to be the potential features of classifiers predicting tau burdens. In particular, different models with various combinations of biomarkers are needed because not all cohorts/centres have access to all biomarkers.

In the present study, we aimed to develop a model to predict tau burdens in the prodromal AD using multimodal biomarkers. We hypothesized that ML could provide an objective, unbiased estimator for classifying tau positivity as an alternative statistical method. We developed and validated several RF and GBM models with various combinations of variables in order to account for the various clinical environments. Variable importance and partial dependency plot (PDP) were also assessed to identify the most relevant features and their relationship to tau burden.

## Results

### Demographics and clinical characteristics of participants

The demographic information of the participants is summarised in Table 1. The A + T + group had a higher percentage of female participants examined compared to the A + T− group (58.8% vs. 26.7%, *p* = 0.009). The A + T− group showed a higher number of years of education than the A + T + group (16.6 ± 3.1 years vs. 15.3 ± 2.1 years, *p* = 0.045). There were no differences in age (p = 0.463) and frequency of APOE4 carriers (p = 0.374) between the A + T− and A + T + groups.

**Table 1:** Demographics and biomarkers of A + MCI participants.

	Total (N = 64)	
	A + T− (N = 30)	A + T + (N = 34)
**Demographics**		
Age, years	72.1 ± 7.2	70.8 ± 7.4
Sex, % female*	8 (26.7%)	20 (58.8%)
Education, years*	16.6 ± 3.1	15.3 ± 2.1
MCI stage, % late MCI	11 (36.7%)	16 (47.1%)
MMSE	26.9 ± 3.9	24.7 ± 5.0
ADAS-cog13*	19.7 ± 8.8	28.3 ± 8.4
**Biomarkers**		
APOE4 carriers	19 (60.3%)	25 (73.5%)
HV mm3*	5776.8 ± 865.0	4854.7 ± 1077.0
AV45 PET SUVR	1.33 ± 0.16	1.44 ± 0.23
Cth_Frontal mm*	3.15 ± 0.12	3.04 ± 0.15
Cth_Temporal mm*	3.37 ± 0.17	3.21 ± 0.20
Cth_Parietal mm*	3.14 ± 0.16	2.94 ± 0.18
Cth_Cingulate mm*	3.35 ± 0.17	3.24 ± 0.23
Cth_Occipital mm*	3.14 ± 0.18	2.99 ± 0.21
Cth_Global mm*	3.19 ± 0.13	3.04 ± 0.16

*MCI* mild cognitive impairment, *MMSE* mini-mental state examination, *PET* Positron emission, *ADAS-cog* The Alzheimer's disease assessment scale-cognitive, *HV* hippocampal volume, *Cth* cortical thickness, *A* amyloid, *T* tau.

*P < 0.05 between A + T− and A + T +.

The A + T + group showed a lower hippocampal volume (4854.7 ± 1077.0 mm^3^ vs. 5776.8 ± 865.0 mm^3^, *p* < 0.001) and decreased cortical thickness in all lobes (*p* = 3.2 × 10^–5^ to 0.042) compared to the A + T− group. The A + T + group also showed a higher score on The Alzheimer’s Disease Assessment Scale-Cognitive (ADAS-Cog) 13-item scale than the A + T− group (28.3 ± 8.4 vs. 19.7 ± 8.8, *p* < 0.001).

### Model performance to classify tau positivity

**Figure 1:**
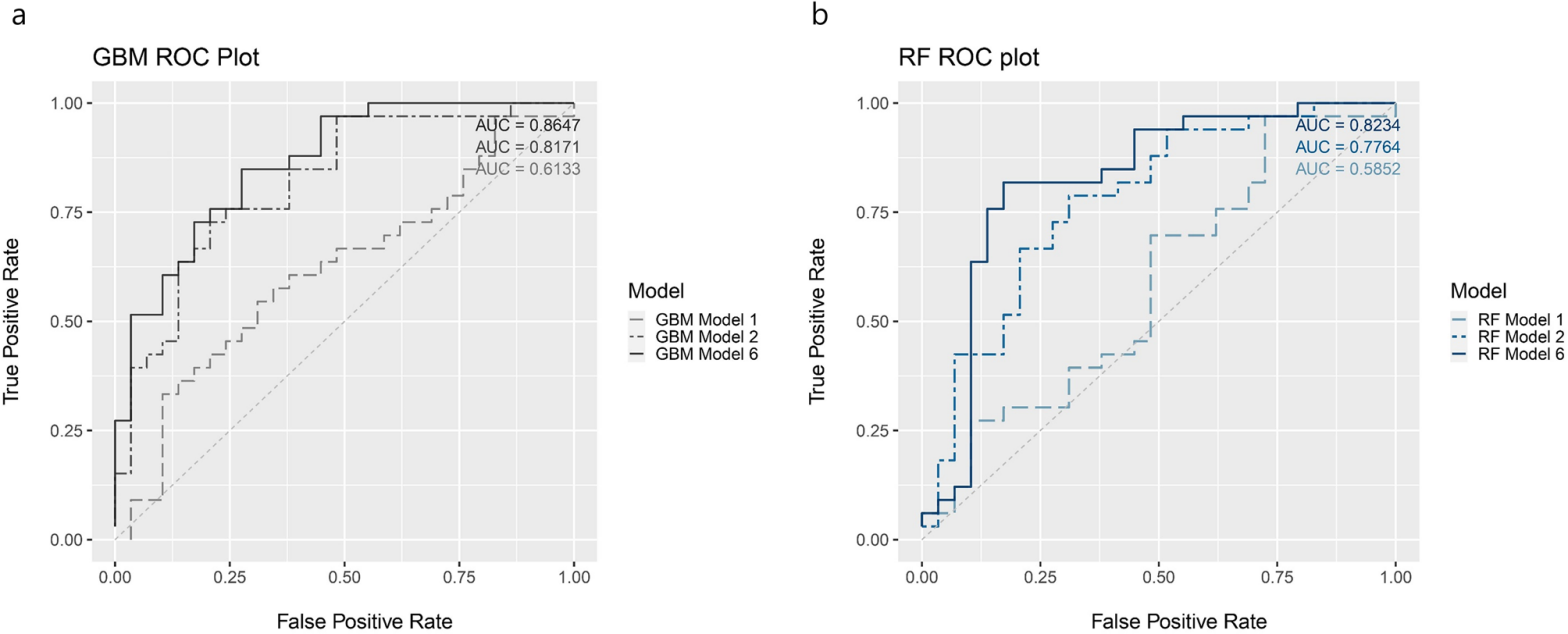
The ROC curve of the proposed method (**a**) GBM (**b**) RF. *ROC* receiver operating characteristic, *AUC* area under the curve, *GBM* gradient boosting machine, *RF* random forest. model 1 = Baseline (Age, Sex, Education) + MCI stage; model 2 = Baseline + MCI stage + NP test; model 6 = Baseline + MCI stage + NP test + APOE4 + cortical thickness. Each plot was made using the R software.

Table 2 presents the performance metrics of GBM and RF for the six different models. The GBM resulted in an AUC of 0.61 (95% CI 0.579–0.647) with the baseline model 1 and a higher AUC of 0.817 (95% CI 0.804–0.830) with model 2 that included NP variables. The highest AUC was 0.86 (95% CI 0.839–0.885) achieved with model 6 (Fig. 1a). The RF had an AUC of 0.59 (95% CI 0.562–0.608) with model 1, 0.77 (95% CI 0.758–0.795) with model 2 and the highest AUC of 0.82 (95% CI 0.808–0.839) using model 6 (Fig. 1b).

**Table 2:** ML models with different combinations of biomarkers using the total dataset.

Model		GBM			RF		
		AUC	95% CI		AUC	95% CI	
Model 1	Baseline (age, sex, education) + MCI stage	0.613375	0.57972	0.64703	0.585162	0.562303	0.608021
Model 2	Baseline + MCI stage + NP test	0.817137	0.803992	0.830282	0.776385	0.758101	0.794668
Model 3	Baseline + MCI stage + NP test + APOE4	0.814002	0.795381	0.832623	0.818182	0.80336	0.833003
Model 4	Baseline + MCI stage + NP test + APOE4 + suvr_fdg	0.867294	0.849891	0.884696	0.801463	0.790315	0.812611
Model 5	Baseline + MCI stage + NP test + APOE4 + HV/ICV	0.825496	0.807022	0.843971	0.792059	0.771281	0.812836
Model 6	Baseline + MCI stage + NP test + APOE4 + cortical thickness	0.862069	0.839286	0.884852	0.823407	0.807749	0.839064

*AUC* Area under the curve, *MCI* mild cognitive impairment, *APOE4* Apolipoprotein E 4 genotype, *NP* Neuropsychological, *GBM* gradient boosting machine, *RF* random forest, *suvr_fdg* Standardized uptake value ratio of fluorodeoxyglucose-positron emission tomography, *HV/ICV* Hippocampal volume/Intracranial volume.

### The relative variable importance and PDP

**Figure 2:**
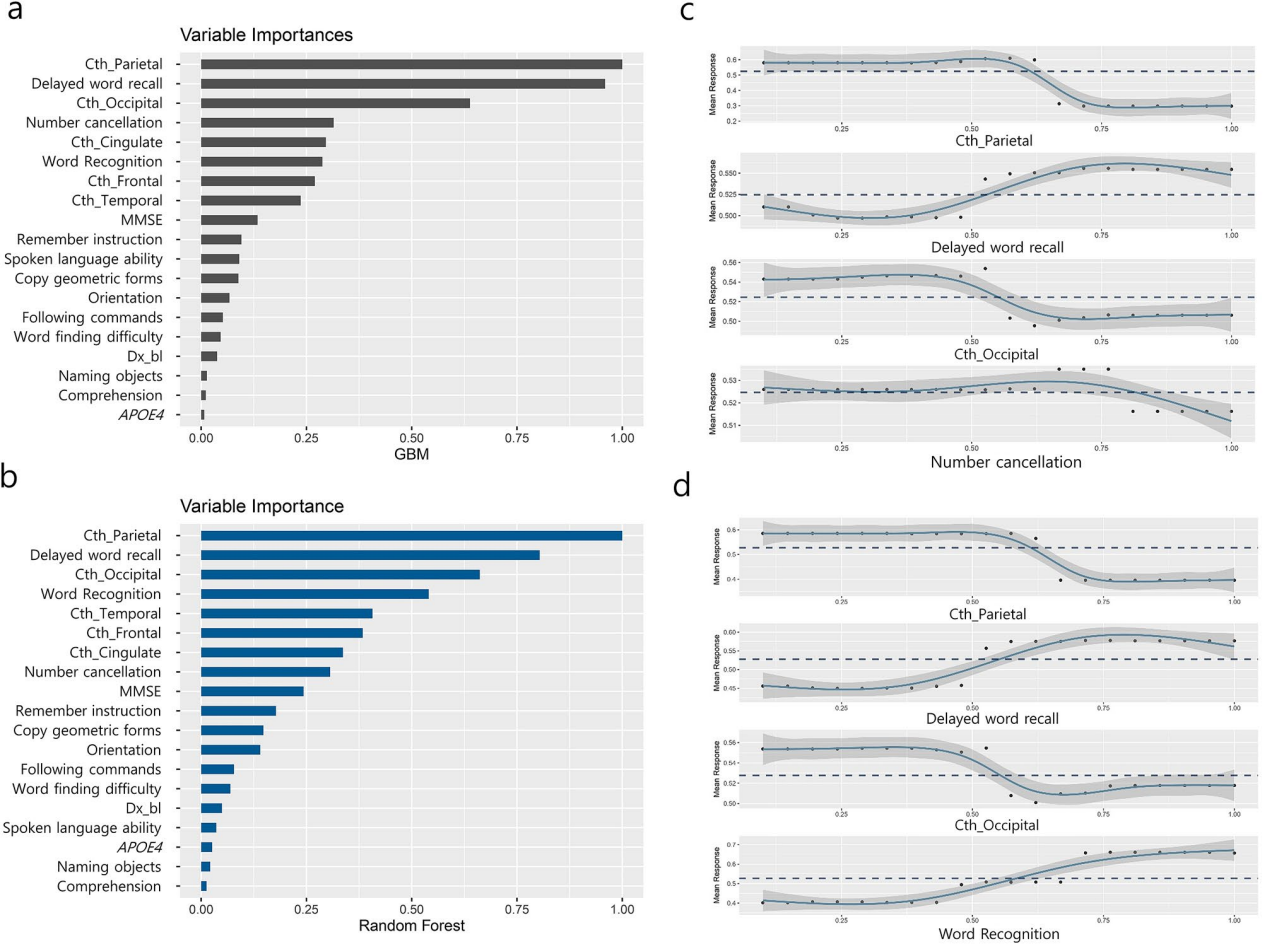
Variable importance plot for model 6 (**a**) GBM and (**b**) RF; and partial dependence plot for model 6 (**c**) GBM and (**d**) RF. *MMSE* mini-mental state examination, *APOE4* Apolipoprotein E 4 genotype, *Cth* cortical thickness, *GBM* gradient boosting machine, *RF* random forest. Each plot was made using the R software.

The relative feature importance from each predictor of model 6 is shown in Fig. 2, indicating the highest contribution to the prediction of tau positivity. In the GBM model, cortical thickness of the parietal lobe was the most important feature followed by the neuropsychological test of memory domains, cortical thickness of the occipital lobe, and number cancellation test score. The important features identified by RF were similar to those identified in the GBM model, such as the cortical thickness of the parietal lobe, the neuropsychological test of memory domains, cortical thickness of the occipital lobe, and word recognition score. As expected, according to the PDP plot, cortical thickness and memory scores are negatively related to the tau accumulation. Additional details regarding the influential variable ranking through model 2 to model 6 are included in Supplementary Table S1.

## Discussion

In the present study**,** we developed and compared ML approaches for prediction of the brain tau burden in prodromal AD patients using multimodal biomarkers based on the ADNI dataset. We found that the GBM with multi-model biomarkers showed a good predictive performance. Especially, the important features in predicting the brain tau burden in prodromal AD patients involved brain structures and neuropsychological results that are responsible for memory. We also found that the GBM with baseline demographics and neuropsychological results showed a reasonable predictive performance. Furthermore, the RF had performance similar to that of the GBM. Therefore, our approaches predicting the tau burden may provide important information for the recruitment of participants in potential clinical trials of tau-targeting therapies, which is helpful to reduce failure in screening. We have developed six models for tau positivity with various combinations of input features reflecting the clinical practice. To construct a model relying on prediction performance alone may lead to underestimating the cost of acquisition and accessibility of the clinical resources. Thus, models from this study maximize the potential applicability of our models in any medical conditions and possibly provide efficient use for deploying cost-effective interventions.

We found that 53.1% of patients with prodromal AD showed a significant tau uptake, which was consistent with that seen in previous studies. Previously, Ossenkoppelle et al. set tau PET-positive (61.4%) as a Youden Index derived cut-off^16,17^ and Maass et al. set significant tau PET uptake as Braak ROI-based staging using a regression-based conditional interference tree approach^8^. Especially in the ADNI data, a significant tau uptake in prodromal AD with conditional inference method analysis was similar (57.9%) to our result.

In the present study, our algorithm using demographics, neuropsychological results, APOE4 genotype, *SUVR of FDG PET* and cortical thickness showed a good predictive performance for predicting tau burden in Aβ + MCI populations. Previously, a study predicting A/T/N stages for a spectrum of individuals ranging from healthy controls to those with MCI and AD was published^18^. This study used structural-MRI alone and showed that the model predicted tau at 89% across the clinical diagnostic group. However, those prediction values were analysed in a healthy control to MCI and AD whereas we did it in a group of homogeneous patients, which could affect predictive performance. Furthermore, in the present study, the GBM with only baseline demographics and neuropsychological results showed a reasonable predictive performance.

The GBM and RF had an adequate performance predicting tau positivity. In the GBM method, a number of weak learners were combined to decrease bias. They generally showed a better performance with low variance data. Since our data set consists of only prodromal AD patients indicating a low variance, the performance of the GBM might be better than RF. In addition, interpretability, which is one of the main challenges of ML, was enabled by providing additional information on the model via variable importance and PDP. The results of this study provide evidence to consider ML to be a more accessible prediction tool for clinical use.

Through analysis of the impact order of the variables in each machine learning classifier, abnormalities in the cortical thickness and neuropsychological tests related to memory function were selected as important features. Our findings were consistent with a recent study showing strong relationships between increased tau pathology and reduced cortical thickness with worse performance on neuropsychological test pronounced in bilateral temporoparietal regions in prodromal AD and AD dementia^15^. Considering that our participants consisted of those with prodromal AD, our findings might be explained by the fact that memory is affected early during the course of AD^19^. Interestingly, we found that the cortical thickness in the occipital region has a strong predictive value for disease severity in prodromal AD. Our findings might be supported by a previous study showing that cognitive function in prodromal/early stage of AD is related to occipital connectivity^20^.

We were able to conduct this study because of the availability of various clinical data through the ADNI because the ADNI is a large cohort of well-characterised subjects, and the clinical and imaging data were based on standardised protocols and analyses. However, there are a few limitations to this study. First, we set binary limits to tau burden as only tau positivity, defined as positive when the in-vivo Braak stage was ≥ III/IV, which is of particular interest since it might be considered as the transitional stage towards AD^8^. There is, however, no consensus yet on how to label tau PET scans as normal or abnormal^8^. However, the frequency of tau (+) in prodromal AD patients^8,17^ seemed to be similar to that observed in previous studies. Second, some etiologically important variables or risk factors that have previously been established in AD research were not examined. Future research should certainly take into account other variables found to be of etiological significance.

Another limitation of our study is relatively small number of samples. There has been no consensus on the measure to estimate the effective sample size for machine learning models. Additionally, acquisition for disease-specific data is still limited and relatively small in clinical practice. Therefore, our result needs to be addressed and clarify using a larger sample size in future studies. Despite these limitations, machine learning with the rigorously well-defined framework proposed here may be useful to explore the nature of heterogeneous tau pathology in the prodromal stage of AD and to examine the relationship between clinical information, neuropsychological profiles, and brain imaging. Developing a better understanding of the algorithms and integration of machine learning into clinical practice is therefore a critical step to support the development of general population prediction models in the prodromal stage of AD.

In conclusion, our ML algorithms for predicting the brain tau burden in prodromal AD showed good accuracy, it can be a useful tool to screen study populations for targeted tau therapies and predict disease severity and prognosis. Future studies are warranted to evaluate tau burden in the transitional stage and account for other significant etiological variables.

## Methods

### Participants

Our study population primarily consisted of subjects from the Alzheimer’s Disease Neuroimaging Initiative (ADNI)-3. A full list of inclusion/exclusion criteria is described in detail at http://adni.loni.usc.edu/methods/documents/. All participants provided written informed consent, and all protocols were approved by each participating site’s institutional review board. The authors obtained approval from the ADNI Data Sharing and Publications Committee for data use and publication. In addition, all methods were implemented in accordance with the approved guidelines. Briefly, MCI participants had a subjective memory complaint with a Clinical Dementia Rating (CDR) score of 0.5 (Petersen et al., 2010). The stage of MCI (early and late) patients was determined using the Wechsler Memory Scale (WMS) Logical Memory II; Early MCI (EMCI) subjects must have education-adjusted scores between approximately 0.5 and 1.5 SD below the mean of cognitively normal adults (on delayed recall of one paragraph from WMS Logical Memory II). All subjects gave written informed consent prior to participation^6^.

**Figure 3:**
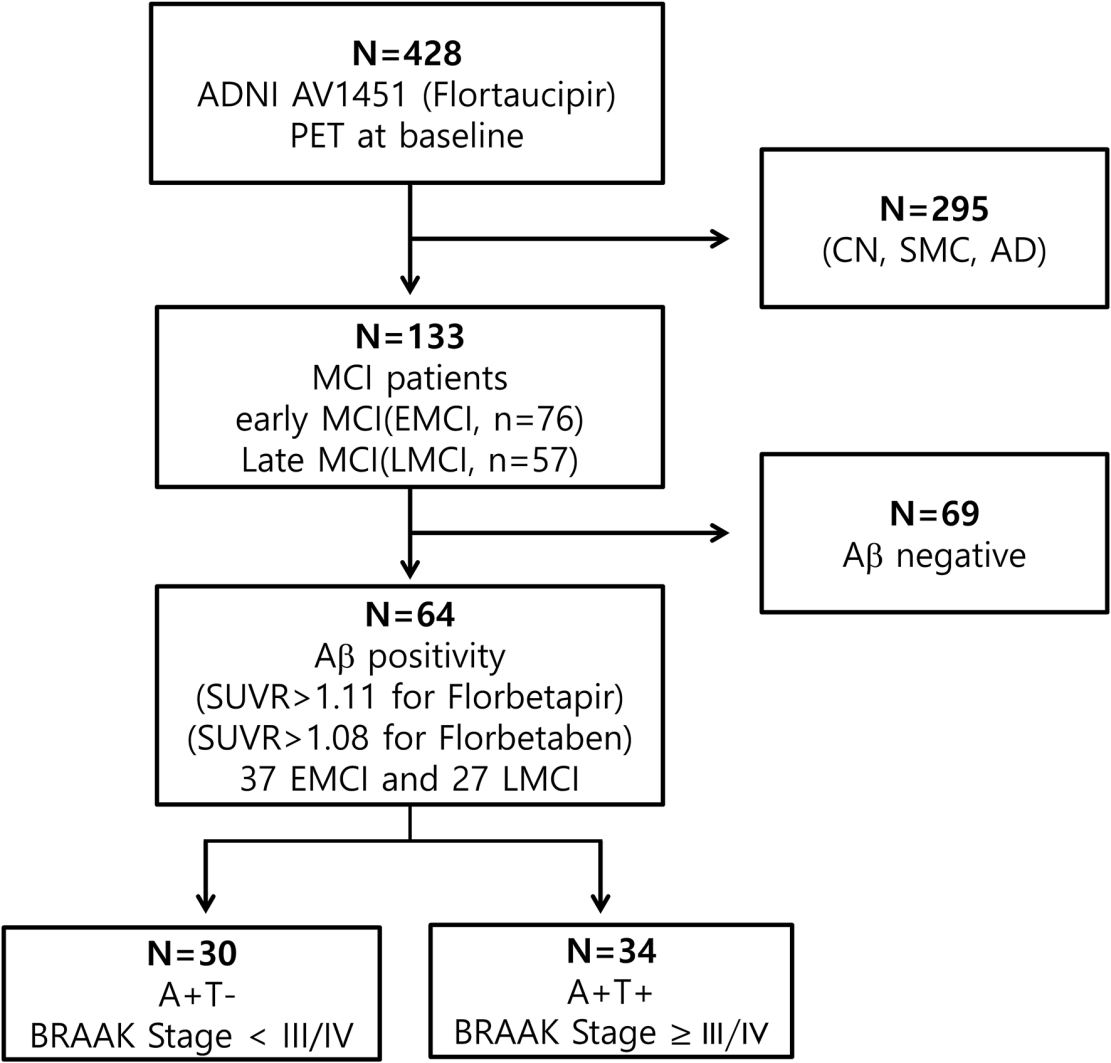
Flowchart showing selection of participants included in the study. *N* number, *CN* cognitively normal, *SMC* subjective memory complaints, *MCI* mild cognitive impairment, *PET* Positron emission tomography, *SUVR* Standardised uptake value ratio, *AD* Alzheimer’s dementia, *A* amyloid, *T* tau.

In this study, we included MCI patients who underwent 3.0 T MRI scanning, ^18^F-AV45 (florbetapir) PET, and AV1451 (flortaucipir) PET at baseline. As of March 2019, a total of 133 patients met this qualification, and their baseline diagnoses were EMCI (n = 76) and late MCI (LMCI, n = 57). Among these, we included in the present study patients with Aβ positivity on AV45 PET, which was defined as standardised uptake value ratios (SUVR) above a cut-off value of 1.11 (Landau et al., 2013; Landau et al., 2012) (37 with EMCI and 27 with LMCI) (Fig. 3).

### Clinical data collection: feature space

Basic demographics and clinical data were extracted from the ADNIMERGE dataset from the ADNI database (http://adni.loni.usc.edu/) in March 2019. Extracted clinical data included the presence of the APOE4 genotype, hippocampal volume (HV), total intracranial volume (ICV), ^18^F-fluorodeoxyglucose (FDG) standardised uptake value ratio (SUVR) (Average FDG SUVR of bilateral angular, inferior temporal, and posterior cingulate regions; AD signature regions relative to the pons/vermis reference region) (Landau et al., 2011a; Landau et al., 2010), AV45 SUVR (Average AV45 SUVR of the frontal, anterior cingulate, precuneus, and parietal cortex relative to the cerebellum) and AV1451 SUVR(Average AV1451 Braak I/II, Braak III/IV and Braak V/VI). The detailed protocols for image processing have been described in previous studies (Bittner et al., 2016; Hsu et al., 2002; Landau et al., 2011b) and in the ADNI methods section at http://adni.loni.usc.edu/.

### Definition of Tau abnormality: outcome

We defined participants as having an abnormal “T” (T +) if their in-vivo Braak stage was III/IV or greater by a conditional inference tree approach. This approach embeds decision tree-structured regression models to determine in-vivo Braak staging based on AV1451 uptake, as suggested by a previous study^21^. The regression model assigned participants with a mean Braak V/VI ROI AV1451 SUVR > 1.267 to in-vivo Braak stage V/VI. The remaining participants underwent the same procedure, using first Braak III/IV (> 1.207) and then Braak I/II (> 1.142) ROIs, leaving the remaining participants in in-vivo Braak stage 0. This conditional inference tree approach thus classified all participants into either Braak V/VI, Braak III/IV, Braak I/II or Braak stage 0 groups.

### Cortical thickness measurement

In order to obtain local cortical thickness measurements for each subject, all T1 volume scans were processed by the CIVET pipeline (version 2.1.0) developed at the Montreal Neurological Institute for fully automated structural image analysis. In brief, using a linear transformation, native MRI images were registered to the MNI-152 template^22^. The N3 algorithm was used for correction of intensity non-uniformity caused by the inhomogeneities in the magnetic field. The next step is to perform the tissue classification into white matter (WM), grey matter (GM), cerebrospinal fluid (CSF), and background (BG) based on the T1-weighed image. The brain is split into the left and right hemispheres for the purpose of surface extraction. The surfaces of the inner and outer cortices were automatically extracted using the Constrained Laplacian-based Automated Segmentation with Proximities (CLASP) algorithm^23^. The inner and outer surfaces had the same number of vertices, and there was a close correspondence between the counterpart vertices of the inner and outer cortical surfaces^24^. The cortical thickness was defined as the Euclidean distance between the linked vertices of the inner and outer surfaces; there were 40,962 vertices in each hemisphere in the native space^23,25^.

Cortical thickness values were calculated in native brain spaces rather than in Talairach spaces because of the limitations of linear stereotaxic normalisation^26^. Intracranial volume (ICV) is defined as the total volume of grey matter, white matter, and cerebrospinal fluid. We calculated ICV by measuring the total volume of the voxels within the brain mask^27^. Brain masks were generated using the FMRIB (Functional Magnetic Resonance Imaging of the Brain) Software Library (FSL) bet algorithm. Since cortical surface models were extracted from MRI volumes transformed into stereotaxic space, cortical thickness was measured in the native space by applying an inverse transformation matrix to the cortical surfaces and reconstructing them in native space^25^.

To measure hippocampal volume (HV), we used an automated hippocampus segmentation method using a graph cut algorithm combined with an atlas-based segmentation and morphological opening as described in an earlier study^28^.

### Machine learning algorithms

To examine changes in prediction accuracy according to the different combinations of predictors, we developed six models. We derived two tree-based ML algorithms: GBM and RF. GBM^29^ is a tree ensemble model that generates a strong prediction model from weak learners, typically decision trees. The RF was proposed by Breiman^30^ builds a tree ensemble predictor with multiple decision trees, in which the predictions of multiple trees are aggregated by averaging or majority voting^31^.

K-fold CV is used to divide the data set into non-overlapping K partitions. K-1 data partitions are used as a training set where a classifier is trained, and its generalization performance is tested on the one left-out validation set. This process is repeated K times. We selected K = 10 as an empirically ideal situation since accuracy is saturated when K = 10. Under the CV procedure, the generalization of the predictive power and validation error was computed. The predictive performance was estimated using the area under the receiver operating characteristic (ROC) curve (AUC) and their 95% confidence interval.

### Interpretable ML: variable importance and partial dependence plot (PDP)

For each optimized model examined the variable importance criterion, which measures the relative prediction power (prediction strength) by using mean decreased accuracy (MDA) or Gini index^10^. For each analysis, variable importance was estimated to find which independent variables were influential features for an accurate classification^32^. Influential variables were ranked by calculating relative importance values. In the tree-based model such as GBM and RF, when the variables split the tree, the relative importance value of that variable was estimated by the discrepancy of the squared error loss over all trees. A higher relative importance value indicated a greater influence of the variables for classifying tau positivity.

We conducted a PDP proposed by J.H. Friedman, which can provide information on whether the feature is positively or negatively correlated to the final prediction. In order to avoid over-weighted or underweighted results, a Min–Max normalisation^33^ was conducted. PDP is a graphical representation tool, which can provide information on whether the feature is positively or negatively related to the final prediction, it is shown as follows.

Let ${{\varvec{x}}}_{{\varvec{s}}}$ be the space of input variables consisting of a chosen subset space and ${{\varvec{x}}}_{{\varvec{c}}}$ be the complemental space,

${{\varvec{x}}}_{{\varvec{s}}}\cup {{\varvec{x}}}_{{\varvec{c}}}=\mathbf{x}$ Then the functional form of approximation $\widehat{f}(\mathbf{x})$ depends on both subset space

$$\widehat{{\varvec{f}}}(\mathbf{x})=\boldsymbol{ }\boldsymbol{ }\widehat{{\varvec{f}}}\boldsymbol{ }\left({{\varvec{x}}}_{{\varvec{s}}\boldsymbol{ }},{{\varvec{x}}}_{{\varvec{c}}}\right),\boldsymbol{ }\boldsymbol{ }\boldsymbol{ }\boldsymbol{ }{\widehat{{\varvec{f}}}}_{{\varvec{c}}}\left({{\varvec{x}}}_{{\varvec{s}}}\right)=\boldsymbol{ }\boldsymbol{ }\boldsymbol{ }\widehat{{\varvec{f}}}\left({{\varvec{x}}}_{{\varvec{s}}\boldsymbol{ }}|{{\varvec{x}}}_{{\varvec{c}}}\right)$$

If the dependency of the complemental space is not too strong, the average function

$${\stackrel{-}{{\varvec{f}}}}_{{\varvec{s}}}\left({{\varvec{x}}}_{{\varvec{s}}}\right)=\boldsymbol{ }\boldsymbol{ }{{\varvec{E}}}_{{{\varvec{x}}}_{{\varvec{c}}}}\left[\widehat{{\varvec{f}}}(\mathbf{x})\right]=\int \widehat{{\varvec{f}}}\boldsymbol{ }\left({{\varvec{x}}}_{{\varvec{s}}\boldsymbol{ }},{{\varvec{x}}}_{{\varvec{c}}}\right){{\varvec{p}}}_{{\varvec{c}}}\left({{\varvec{x}}}_{{\varvec{c}}}\right){\varvec{d}}{{\varvec{x}}}_{{\varvec{c}}}$$

where ${p}_{c}\left({x}_{c}\right)$ is a marginal probability density function of ${x}_{c}$.

An alternative functional form of approximation $\widehat{f}(\mathbf{x})$ becomes

$${\stackrel{\sim }{{\varvec{f}}}}_{{\varvec{s}}}\left({{\varvec{x}}}_{{\varvec{s}}}\right)=\boldsymbol{ }\boldsymbol{ }{{\varvec{E}}}_{\mathbf{x}}[\widehat{{\varvec{f}}}(\mathbf{x})|{{\varvec{x}}}_{{\varvec{s}}}]=\int \widehat{{\varvec{f}}}(\mathbf{x}){{\varvec{p}}}_{{\varvec{z}}}\left({{\varvec{x}}}_{{\varvec{c}}}|{{\varvec{x}}}_{{\varvec{s}}}\right){\varvec{d}}{{\varvec{x}}}_{{\varvec{c}}}$$

### Statistical analysis

For the comparison of demographic and clinical data, a two-sample t-test was used for continuous variables, and a chi-square test was used for categorical variables. All analyses were performed with R package^34^, version 3.6.1 (R Project for Statistical Computing).

## Supplementary Information

Supplementary Information

## Data Availability

The datasets used and/or analysed during the current study are available from the corresponding author on reasonable request.
