# Supplementary material for: Prediction of tau accumulation in prodromal Alzheimer’s disease using an ensemble machine learning approach
Source: Sci Rep. 2021 Mar 11;11:5706. doi: 10.1038/s41598-021-85165-x (PMC7970986; doi:10.1038/s41598-021-85165-x)
Supplement: Supplementary file 1 — Supplementary Information [file 41598_2021_85165_MOESM1_ESM.docx]

**Prediction of tau accumulation in prodromal Alzheimer’s disease using an ensemble** **machine learning approach**

Jaeho Kim, MD, PhD^1^, Yuhyun Park, BS^2,3^, Seongbeom Park, MS^2^, Hyemin Jang, MD, PhD^2,4,5^, Hee Jin Kim, MD, PhD^2,4,5^, Duk L. Na, MD, PhD^2,4,5,6,7^, Hyejoo Lee^2,4,5*^, Sang Won Seo, MD, PhD^2,3,4,5,7*^

^1^Department of Neurology, Dongtan Sacred Heart Hospital, Hallym University College of Medicine, Hwaseong-si, Gyeonggi-do, Republic of Korea

^2^Department of Neurology, Samsung Medical Center, Sungkyunkwan University School of Medicine, Seoul, Republic of Korea

^3^Department of Intelligent Precision Healthcare Convergence, Sungkyunkwan University School of Medicine, Suwon, Korea

^4^Neuroscience Center, Samsung Medical Center, Seoul, Korea

^5^Samsung Alzheimer Research Center, Samsung Medical Center

^6^Stem Cell & Regenerative Medicine Institute, Samsung Medical Center

^7^Department of Health Sciences and Technology, SAIHST, Sungkyunkwan University, Seoul, Republic of Korea

* These authors contributed equally to this work as co-corresponding authors

**Corresponding author 1: Sang Won Seo, MD, PhD**

Department of Neurology, Samsung Medical Center, Sungkyunkwan University School of Medicine, 81 Irwon-ro, Gangnam-gu, Seoul, 06351, Republic of Korea

Phone: +82-2-3410-1233 Fax: +82-2-3410-0052,

E-mail: sangwonseo@empas.com

**Co-Corresponding author 2: Hyejoo Lee, PhD**

Department of Neurology, Samsung Medical Center, Sungkyunkwan University School of Medicine, 81 Irwon-ro, Gangnam-gu, Seoul, 06351, Republic of Korea

Phone: +82-2-3410-1233 Fax: +82-2-3410-0052,

E-mail: hyejoo271@gmail.com

**Table S1.** Impact order of variables in each GBM/RF model

1. Highly ranked variables by GBM

| Model 2 | | Model 3 | | Model 4 | | Model 5 | | Model 6 | |
| --- | --- | --- | --- | --- | --- | --- | --- | --- | --- |
| Variable | RI | Variable | RI | Variable | RI | Variable | RI | Variable | RI |
| Q4 score | 195.4 | Q4 score | 148.2 | Q4 score | 164.7 | Q4 score | 180.3 | Cth Parietal | 129.3 |
| Q8 score | 92.9 | Q8 score | 115.1 | Q8 score | 114.8 | Q8 score | 88.3 | Q4 score | 124.0 |
| Q13 score | 65.1 | Q13 score | 69.8 | FDG | 63.4 | HV | 81.9 | Cth Occipital | 82.5 |
| Q3 score | 40.5 | MMSE | 49.0 | Q13 score | 62.1 | FDG | 60.5 | Q13 score | 40.7 |
| MMSE | 38.4 | Q3 score | 33.4 | Q3 score | 35.2 | Q13 score | 52.4 | Cth Cingulate | 38.3 |
| Q7 score | 23.7 | Q7 score | 27.5 | MMSE | 33.7 | MMSE | 26.7 | Q8 score | 37.2 |
| Q2 score | 16.1 | Q2 score | 20.2 | Q2 score | 16.7 | Q3 score | 24.0 | Cth Frontal | 34.9 |
| Dx bl | 13.3 | Dx bl | 12.1 | Dx bl | 11.9 | Q7 score | 13.1 | Cth Temporal | 30.6 |
| Q11 score | 8.7 | ApoE4 | 10.0 | Q7 score | 11.4 | Q2 score | 12.9 | MMSE | 17.3 |
| Q5 score | 6.3 | Q11 score | 8.4 | ApoE4 | 7.8 | Dx bl | 8.6 | Q9 score | 12.4 |

1. Highly ranked variables by RF

| Model 2 | | Model 3 | | Model 4 | | Model 5 | | Model 6 | |
| --- | --- | --- | --- | --- | --- | --- | --- | --- | --- |
| Variable | RI | Variable | RI | Variable | RI | Variable | RI | Variable | RI |
| Q4 score | 910.0 | Q4 score | 912.1 | Q4 score | 815.4 | Q4 score | 818.6 | Cth Parietal | 865.9 |
| Q8 score | 862.6 | Q8 score | 769.8 | Q8 score | 709.3 | Q8 score | 765.7 | Q4 score | 696.2 |
| MMSE | 340.4 | MMSE | 369.1 | FDG | 473.6 | HV | 605.1 | Cth Occipital | 573.0 |
| Q13 score | 310.1 | Q13 score | 261.8 | Q13 score | 292.8 | FDG | 373.8 | Q8 score | 468.0 |
| Q7 score | 233.5 | Q7 score | 209.5 | MMSE | 288.6 | Q13 score | 290.4 | Cth Temporal | 352.3 |
| Q3 score | 182.9 | Q3 score | 192.6 | Q7 score | 189.6 | MMSE | 276.3 | Cth Frontal | 332.2 |
| Q2 score | 113.9 | Q2 score | 118.6 | Q3 score | 170.5 | Q7 score | 158.6 | Cth Cingulate | 291.6 |
| Q9 score | 100.5 | Q9 score | 118.6 | Q2 score | 89.4 | Q3 score | 146.6 | Q13 score | 265.3 |
| Dx bl | 85.2 | Q11 score | 69.1 | Q9 score | 85.2 | Q2 score | 91.9 | MMSE | 210.6 |
| Q11 score | 56.2 | Dx bl | 62.3 | ApoE4 | 51.6 | Q9 score | 84.4 | Q9 score | 153.9 |

Abbreviations: MMSE, mini-mental state examination; APOE, apolipoprotein e; Cth, cortical thickness; Q1, word-recall Trials; Q2, following commands; Q3, copy geometric forms; Q4, delayed word recall; Q5, naming objects; Q6, following instructions; Q7, orientation; Q8, word recognition; Q9, remember instruction; Q11, word-finding difficulty; Q13, number cancellation; GBM, gradient boosting machine; RF, random forest
